# Intrinsic K-Ras dynamics: A novel molecular dynamics data analysis method shows causality between residue pair motions

**DOI:** 10.1038/srep37012

**Published:** 2016-11-15

**Authors:** Sezen Vatansever, Zeynep H. Gümüş, Burak Erman

**Affiliations:** 1 Department of Chemical and Biological Engineering, College of Engineering, Koç University, Rumelifeneri Yolu, 34450, Sarıyer, Istanbul, Turkey; 2 Department of Genetics and Genomics, Icahn School of Medicine at Mount Sinai, New York, NY 10029, USA; 3 Icahn Institute for Genomics and Multiscale Biology, Icahn School of Medicine at Mount Sinai, New York, NY 10029, USA.

## Abstract

K-Ras is the most frequently mutated oncogene in human cancers, but there are still no drugs that directly target it in the clinic. Recent studies utilizing dynamics information show promising results for selectively targeting mutant K-Ras. However, despite extensive characterization, the mechanisms by which K-Ras residue fluctuations transfer allosteric regulatory information remain unknown. Understanding the direction of information flow can provide new mechanistic insights for K-Ras targeting. Here, we present a novel approach –*conditional* time-delayed correlations (CTC) – using the motions of all residue pairs of a protein to predict directionality in the allosteric regulation of the protein fluctuations. Analyzing nucleotide-dependent intrinsic K-Ras motions with the new approach yields predictions that agree with the literature, showing that GTP-binding stabilizes K-Ras motions and leads to residue correlations with relatively long characteristic decay times. Furthermore, our study is the first to identify driver-follower relationships in correlated motions of K-Ras residue pairs, revealing the direction of information flow during allosteric modulation of its nucleotide-dependent intrinsic activity: active K-Ras Switch-II region motions drive Switch-I region motions, while α-helix-3L7 motions control both. Our results provide novel insights for strategies that directly target mutant K-Ras.

K-Ras is a small GTP-binding protein pivotal in cellular signaling. Somatic K-Ras mutations are among the most common activating cancer lesions, especially driving pancreas, colon and lung cancers^1^,^2^,^3^. Signaling through K-Ras is dependent on the bound nucleotide, where the GTP-bound state is active while the GDP-bound state is inactive. In GTP-bound K-Ras, P-loop (residues 10–17), switch I (SI, residues 25–40) and switch II (SII, residues 60–74) regions make up the active site whose well-ordered conformations allow effector protein binding for K-Ras signaling (Fig. 1). However, oncogenic gain-of-function mutations impair GTP hydrolysis and freeze K-Ras in its active state^4^, causing uncontrollable cellular growth and evasion of apoptotic signals^5^,^6^,^7^. Tumors driven by oncogenic K-Ras are often resistant to standard therapies and result in poor outcomes; they are also excluded from treatment with other targeted therapies, making mutant K-Ras a high priority target in cancer treatment^8^,^9^. However, no clinically available drugs directly target mutant K-Ras.

Part of the challenge in oncogenic K-Ras inhibitor design has been due to structure analyses that suggest a lack of well-defined druggable sites on its surface^10^. However, studies that have utilized protein dynamics data such as NMR and mass spectrometry have identified binding pockets on specific K-Ras oncogenic mutants and have attempted to stabilize their conformational states^11^,^12^,^13^,^14^. Accumulating studies suggest that K-Ras proteins are in dynamic and flexible states and their distinct characteristics cannot be identified by structural studies alone^12^,^13^,^14^,^15^,^16^,^17^,^18^,^19^,^20^,^21^. K-Ras dynamics in different conformational states, that can also change due to allosteric interactions between protein residues, also need to be quantified^22^. However, we still need to clearly understand the intra-molecular allosteric networks between distant sites on K-Ras^23^. While such allosteric interaction sites have recently been discovered in its catalytic domain^24^,^25^,^26^, they remain largely understudied^23^. Understanding allosteric interactions can present novel opportunities for small molecules that target mutant K-Ras in attempts to restore its dynamics to those of the wild-type, which first requires a deeper understanding of the intrinsic K-Ras dynamics.

In allosteric regulation of protein dynamics, correlated motions between protein residues are essential^27^,^28^,^29^. These motions enable the transfer of fluctuation information through the allosteric network^30^, which inherently involves directionality, or “causality” of events^31^. If the motions of two residues are correlated, it would be valuable to identify whether the motions of one residue drive the motions of the other. However, while correlation calculations indicate interaction (which is necessary for allosteric transitions) they are symmetric and do not reveal the direction of information flow.

Here, we introduce a novel method that predicts causality relationships between residue pairs of a protein. For this purpose, we first record residue fluctuations calculated at every time step of a molecular dynamics (MD) simulation as a time series. We then calculate the *conditional* time-delayed correlation (CTC) of a residue pair as the correlation between two time series subject to the condition that fluctuations of the first trajectory are correlated with later fluctuations of the second and thereby predict how past fluctuations of one trajectory affects the future fluctuations of the second. In some cases, CTC function of two trajectories may be asymmetric, with one affecting the other more strongly. We then predict that the fluctuations of a given residue control and modify the fluctuations of the delayed one. CTC in two time-series is extensively used in causality analyses in economics since its inception^32^, leading to the Nobel prize, but has not been widely adopted in biophysics. This is a new approach to analyzing of structure-function relationships. We demonstrate the simplicity of computing CTC functions in studying protein dynamics by applying it to understand K-Ras motions. We specifically focus on K-Ras because it is clinical important and well-characterized in the literature, enabling us to validate our predictions. While correlations between the fluctuations of residue pairs have already been shown in several Ras protein studies^33^,^34^,^35^, despite extensive literature on K-Ras, there has been little attention on the role of causality (or directionality) in correlation dynamics of its residues.

In summary, we present a comprehensive study of intrinsic K-Ras dynamics, including detailed analyses of causality between the motions of its residues. We first provide detailed, quantitative descriptions of both GTP- and GDP-bound K-Ras from extensive MD simulations. We use a statistical thermodynamics interpretation of fluctuation correlations to quantify K-Ras ‘stiffening’ upon activation. Stiffening changes protein dynamics. More importantly, using stiffness calculations jointly with measurements of reduced relative fluctuations, we define protein stability and show that K-Ras is more stable in active conformation. To characterize correlated motions that are persistent within the MD simulations of GTP- and GDP bound K-Ras, we map the correlated motion patterns within their residues individually, and then compare and discuss their correlation decay time differences in detail. Our results show that inactive K-Ras is marked by a pronounced decrease in correlated motions of residues for shorter periods, while active K-Ras correlations have longer decay times. We analyze the ensuing events at the atomic scale. Finally, to enable a deeper understanding of K-Ras dynamics, we introduce the first causality calculations for K-Ras and predict specific driver/follower residue pairs during protein simulations.

## Results and Discussion

### Comparison of stiffness changes in active and inactive K-Ras

#### GTP binding increases K-Ras stiffness

To understand how nucleotide binding affects K-Ras dynamics, we quantified changes in its ‘stiffness’ – a metric that inversely correlates with residue pair fluctuations - upon GTP vs. GDP binding. For this purpose, we represented the interaction between two fluctuating residue pairs (*i* and *j*) as a spring with a constant *k*_*ij*_, and related its magnitude to the mean square fluctuations of residues *i* and *j* and to their cross-correlations using the Gaussian Network Model (GNM). GNM is a coarse grained model at the residue level but it has been used widely for predicting protein behavior^36^. Plotting this spring constant for every residue pair in both GTP- (Fig. 2A) and GDP-bound (Fig. 2B) K-Ras, we observe strong coordination in the fluctuations of GTP phosphate groups with those of K-Ras (Fig. 2A).

To zoom in on and directly compare the effects of nucleotide binding on K-Ras stiffness, we calculated the differences in spring constant values between GTP- and GDP- bound K-Ras. In the following paragraphs we show that the spring constants calculated in this way are in agreement with experimental findings. In Fig. 2C, red dots indicate that the differences are largely due to the stiffening effects of GTP-binding on residue pair fluctuations. Notice that Regions 1–3 in Fig. 2C that correspond to secondary structures show significant increase in *k*_*ij*_ when GTP-bound. Furthermore, Region 1 corresponds to strong coordination of β2 and β3 motions, while Regions 2 and 3 correspond to increased stiffness of β4-α3 and α4.

#### Nucleotide binding affects spring constant of α2 (SII)

We next investigated the effects of nucleotide binding on the spring constant of α2 (SII), because previous studies have shown that stiffness increases when SII refolds into an α-helical conformation through GTP binding^37^. We calculated the spring constants of the two terminal residues of α2 (A66 and T74), which were 0.10 kcal/mol∙A^2^ (69.91 pN/nm) for active and 0.04 kcal/mol∙A^2^ (27.78 pN/nm) for inactive K-Ras. Previous studies have utilized various experimental methods that have all led to spring constants within ~0.09–1.15 kcal/mol∙A^2^ (60–80 pN/nm) for helices^38^,^39^. Our results for both K-Ras forms are on the same order of magnitude. Note that for active K-Ras the α2 spring constant is equalent to the characteristic spring constant of α-helices, while it is lower in inactive form. Hence, our results validate and quantify earlier, qualitative observations of Noe *et al.*^37^ that the α2 spring constant reaches to the level of an α-helix spring constant during GTP binding.

#### Overall spring constant is higher in active complex

To estimate global changes in stiffness in response to nucleotide binding, we calculated the overall spring constants *k*_*overall*_ (*details in Methods*) of nucleotide-K-Ras complexes, which were 0.70 kcal/mol∙A^2^ (481.75 pN/nm) for GTP-bound, and 0.55 kcal/mol∙A^2^ (385.12 pN/nm) for GDP-bound K-Ras. Both are of the same order of magnitude with an experimental study for another protein, myoglobin, which has an overall spring constant of ~300 pN/m^40^,^41^ pointing to an order of magnitude agreement of overall stiffnesses of proteins in general. In conclusion, GTP-binding increases the overall K-Ras stiffness. In other words, GTP-binding decreases pairwise residue fluctuations of K-Ras overall, making the protein more rigid.

#### Secondary structure motions show the strongest coordination with the rest of the protein

Quantifying the spring constant based on fluctuations allows for analyzing how, analogous to a virtual spring, the fluctuations of a specific residue are coupled with fluctuations of rest of the protein. To discover residues whose fluctuations are in strong coordination with K-Ras fluctuations and how they change between the two states, we compared the mean spring constant 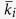 of each residue *i*, for both active and inactive K-Ras (Fig. 2D) as described in *Methods*. A large 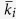 value indicates that the motions of residue *i* are stiffly coupled with protein motions; while a small 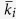 value indicates that the motions of the *i*th residue and the protein are flexibly coupled. For simplicity, we categorized the significant mean spring constant 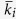 values as highest, high and smallest (For details please see Supplementary Table S1). In both states, the highest 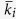 values are of β-strand residues β4, β5 and β6, showing the strongest coordination of their motions with K-Ras motions. Next, high 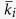 values of β1, P-loop and α5 residues indicate that their fluctuations are also strongly coupled with those of the protein. On the other hand, the smallest 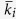 values belong to SII region in active and SI and SII regions in inactive K-Ras which show that their residue fluctuations are not correlated with the rest of the protein (Supplementary Table S2). Since we have defined the stiffness metric as a signifier of a decrease in residue fluctuations, we provide a second line of proof that increased stiffness stabilizes dynamic fluctuations in both forms of K-Ras by using Root Mean Square Fluctuation (RMSF) graph (Supplementary Figure S1). Clearly, the residues with the smallest mean spring constant 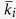 values from Fig. 2D have the highest RMSF values in Supplementary Fig. S1 and vice versa.

**Figure 1:**
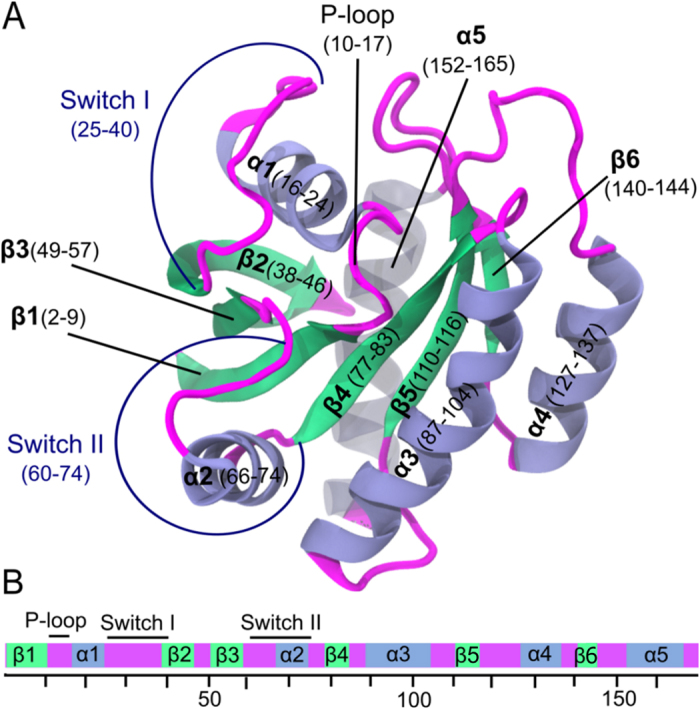
Three-dimensional structure of wild-type K-Ras protein in GTP-bound state (PDB: 4OBE). (**A**) K-Ras structure ribbon representation with secondary structures in blue for α-helices and green for β-sheets. (**B**) Schematic of K-Ras sequences (residues 1–169). Functional regions are in same color used in K-Ras structure in **A**.

As indicated in previous studies where NMR and Atomic Force Microscopy were used, protein stiffness depends on secondary structure^40^,^41^, where loops contribute to structural flexibility and show large fluctuations, while β-strands and α-helices provide mechanical stability and show small fluctuations^40^. Our K-Ras results are consistent with these general observations. In addition, we observe stiff coupling of the fluctuations of the P-loop and the protein. This observation is important since P-loop is the phosphate binding site of K-Ras and connects β1 and α1 (Fig. 1). Although loops are often flexible regions of proteins and show higher fluctuations, in K-Ras, motions of P-loop residues are stiffly coupled to those of the protein, especially in active state (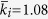 kcal/mol∙A^2^ for K-Ras-GTP, 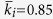 kcal/mol∙A^2^ for K-Ras-GDP).

#### The mean spring constant values of residues in β2, β3, α3 and switch regions –especially SI- are higher in active K-Ras than in inactive K-Ras

**Figure 2:**
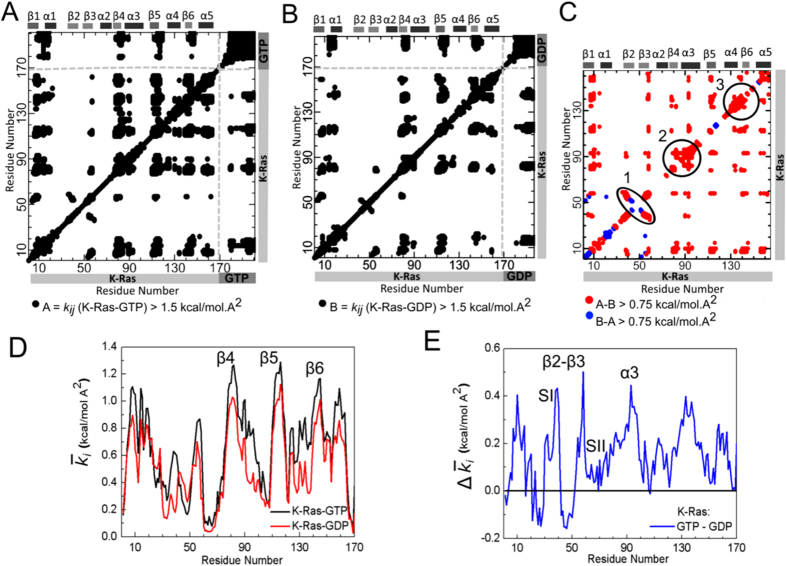
Stiffness results for GTP- and GDP-bound K-Ras and their difference. In panels A and B, both axes marks 1–169 represent the residue Cα atoms of K-Ras and marks 170-on represent GDP and GTP nucleotide heavy atoms, respectively with *k*_*ij*_ > 1.5 kcal/mol∙A^2^. (**A**) *k*_*ij*_ for K-Ras-GTP. Atoms 170–181 are the γ, β, α-phosphate groups and 182–201 are the guanine atoms of GTP. (**B**) *k*_*ij*_ for K-Ras-GDP. Atoms 170–178 are the β and α phosphate groups and 178–197 are the guanine atoms of GDP. (**C**) Difference between active and inactive K-Ras *k*_*ij*_ values. Red regions are stiffer in K-Ras-GTP (*k*_*ij*_values of K-Ras-GTP > K-Ras-GDP by at least 0.75 kcal/mol∙A^2^) and blue regions are stiffer in K-Ras-GDP (*k*_*ij*_values of K-Ras-GDP > K-Ras-GTP by at least 0.75 kcal/mol∙A^2^). (**D**) Mean spring constants 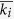 for GTP and GDP bound states. (**E**) Mean spring constant differences 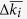
for GTP and GDP bound states. Positive values correspond to larger mean stiffness in K-Ras-GTP.

Finally, we calculated mean spring constant differences between GTP-bound active and GDP-bound inactive K-Ras, 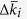 (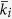
_K-Ras-GTP_  − 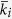
_K-Ras-GDP_). Figure 2E shows that the fluctuations of β2 and β3 terminal (D38 and D57) and α3 center (D92-I93) residues are in stronger coordination with those of active K-Ras (vs. inactive K-Ras) (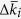 > 0.43 kcal/mol∙A^2^). Our results also indicate that although residues of switch regions have the smallest 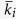 values in both forms, some of their 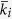 values increase significantly in active form. In Fig. 2E, 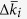
ranges between 0.20–0.36 kcal/mol∙A^2^ for residues in SI (D30-R41) and 0.02–0.19 kcal/mol∙A^2^ for residues in SII (G60-T74). These 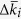 values show stiffer coupling of the motions of GTP-K-Ras with the motions of switch residues, especially SI (vs GDP-K-Ras). This result is important as SI includes the binding site to effector proteins which only bind to GTP-bound K-Ras when SI flexibilty is reduced^42^. Earlier studies that used NMR spectra and RMSF calculation also support our results that GTP binding reduces the flexibility of both SI and SII, especially SI^35^,^43^. Our results improve on this information by showing that fluctuations of switch regions –notably SI- are more stiffly coupled with K-Ras-GTP fluctuations (Fig. 2E).

### Comparison of residue pair correlations for active and inactive K-Ras

**Figure 3:**
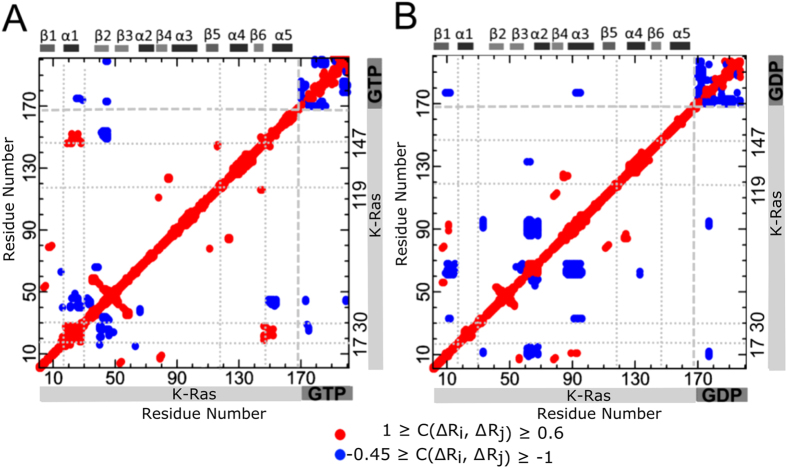
Cross-correlation coefficient maps for GTP and GDP bound states. Red dots show positive correlations (1 ≥ C(Δ*R*_*i*_, Δ*R*_*j*_) ≥ 0.6) and blue dots show negative correlations (−0.45 ≥ C(Δ*R*_*i*_, Δ*R*_*j*_) ≥ −1). Residues indices 1–169 refer to K-Ras. (**A**) Correlated fluctuations of K-Ras-GTP. Indices between 170–201 refer to GTP heavy atoms (182–201 are guanine atoms). (**B**) Correlated fluctuations of K-Ras-GDP. Indices between 170–197 refer to GDP heavy atoms (178–197 are guanine atoms).

To identify if the fluctuations of one residue are related to fluctuations of another residue, we calculated the correlations of all residue-residue pairs in both GTP- vs GDP bound K-Ras complexes. As expected, cross-correlation coefficient maps of K-Ras-GTP (Fig. 3A) and K-Ras GDP (Fig. 3B) exhibit different correlation characteristics. The most remarkable differences between Fig. 3A and B belong to two parts: (i) the correlation of α1-SI with L10-α5 and (ii) the correlations between β2 and β3. Positive correlation patterns within these two parts are evident in K-Ras-GTP simulations, but absent in K-Ras GDP simulations. To provide comprehensive information on nucleotide-dependent K-Ras dynamics, we present these two remarkable results from correlation analyses (Fig. 3) as well as sources of correlated motions (i.e. H-bonds) together in the following sections.

#### The correlation of α1-SI with L10-α5 in active K-Ras motions is due to three specific H-bonds

MD simulations show that the correlation between α1-SI and L10-α5 in the active form results from GTP binding to active site residues, which also form specific H bonds with other K-Ras residues and water. Based on the average number of H-bonds each residue forms throughout the simulation, we estimated that the nucleotides remain bound to active site residues S17, D30, D119 and K147, and that GTP-binding (vs. GDP) is more stable for S17 and D30 (Supplementary Table S3). Furthermore, correlated motions of α1-SI and L10-α5 in GTP-bound K-Ras originate specifically from three H-bonds: (i) A146-Q22, (ii) D30-GTP, (iii) D30-a water molecule. We observed a sustained H-bond between A146-Q22 during active but not in inactive complex simulation. This suggests that A146-Q22 interaction causes a strong relationship between L10α5 (A146-D154) and α1 (L19-I24) in active K-Ras (Fig. 3A) with a correlation coefficient of 0.75, and a weak correlation coefficient of 0.28 for inactive K-Ras. At the same time, the active site residue D30 forms an H-bond with the nucleotide in both active and inactive K-Ras, while it also binds to a water molecule only in the active form. However, the H-bond in the active form between D30(O)-GTP(O2A) is more permanent than the H-bond in the inactive form between D30(O)-GDP(O2’).

#### Since H-bond of D30-GTP is effective throughout the full trajectory, the D30-GTP distance is invariant and the fluctuation correlations of the D30-GTP have longer decay times during K-Ras-GTP simulation

**Figure 4:**
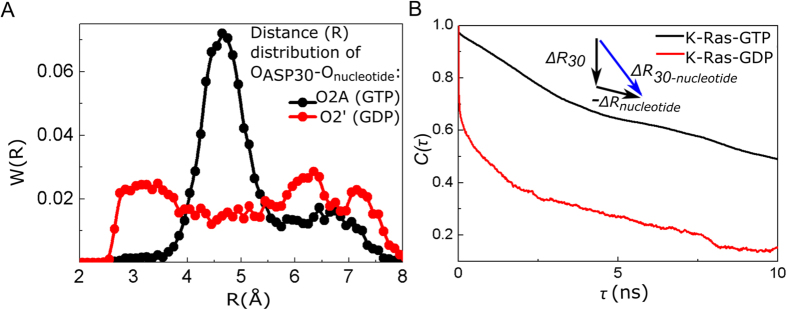
D30-GTP distance is more stable than that of D30-GDP. Fluctuations of D30(O) to GTP(O2A) “connectivity vector” are persistently correlated. (**A**) Distance distribution between D30 and connecting O atoms of GTP (black) and GDP (red) (**B**) Time delayed autocorrelations for the vector connecting Oxygen atom of D30 to O2A of GTP (black curve) and O2’ of GDP (red curve). X-axis is the time delay (*τ*) and Y-axis is the time delayed autocorrelation of the vector for *τ*.

We next combined cross-correlation results with the distance distribution of D30 and nucleotides and quantified the decay times of their correlations during MD simulations. In addition to more permanent binding of D30(O)-GTP(O2A), nucleotide-D30 distance distribution pattern is close to the normal distribution curve with a mean of a smaller value in active K-Ras (Fig. 4A), with a correlation coefficient of 0.97. To quantify decay time of this correlation in both complexes, we first defined two “connectivity vectors”, *ΔR*_*30-GTP*_ and *ΔR*_*30-GDP*_, between D30(O) and nucleotides. As illustrated in Fig. 4B, *ΔR*_*30-GTP*_ connects the starting point of fluctuation vector of *ΔR*_*D30(O*)_ to end point of negative *ΔR*_*GTP(O2A*)_; *ΔR*_*30-GDP*_ starts from *ΔR*_*D30(O*)_ to negative *ΔR*_*GDP(O2’*)_. We then calculated time-delayed autocorrelations of each connectivity vector throughout the MD simulations. The autocorrelation plot in Fig. 4B summarizes the correlation of connectivity vectors at various time delays, where vector correlation coefficients are plotted with 1 ns delays at a time; slow decay of correlations in active K-Ras is clearly observed. Correlations decay to 1/e in about 3 ns for K-Ras-GDP (red line), vs. to ~10 ns for K-Ras-GTP (black line). One reason for this slow correlation decay is the H-bond, which binds D30 to a water molecule in active K-Ras. The O atom of D30 establishes an H-bond with the nearest water during 28% of the trajectory while it does not make any contact with waters when K-Ras is inactive.

#### A continuously acting H-bond stabilizes β2-β3 distance and promotes longer decay times for β2-β3 correlations during K-Ras-GTP simulation

**Figure 5:**
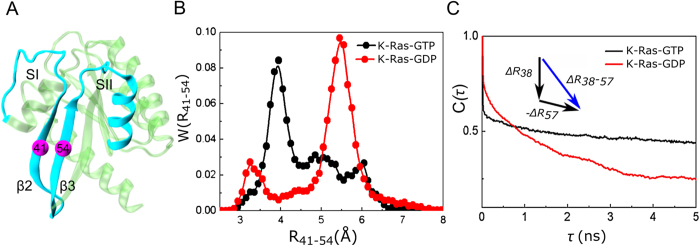
Correlation of β2 and β3 fluctuations is persistent in active K-Ras. (**A**) Locations of R41 (β2) and D54 (β3) relative to SI & SII. (**B**) Distance distribution between Cα atoms of R41 and D54 in K-Ras-GTP (black) and K-Ras-GDP (red). Distance values between R41 (β2) and D54 (β3) populate at 3.90 Å during GTP binding; they populate at 5.46 Å for GDP-bound K-Ras. (**C**) Time delayed autocorrelations for the fluctuations of the vector from D38 (Cα) to D57 (Cα).

β2 and β3 are two parallel β strands located between SI and SII regions (Fig. 5A). Due to the presence of a persistent H-bond between R41(β2)-D54(β3) in K-Ras-GTP simulation, the peak value of *R*_*41–54*_distribution decreases (Fig. 5B) and fluctuations of β2 and β3 become correlated (Fig. 3A). Time-delayed autocorrelations of the vector *ΔR*_*38–57*_ between their terminal residues D38 and D57 are presented in Fig. 5C showing that *ΔR*_*38–57*_correlation decays much more slowly in active K-Ras.

### Causality of Correlated Motions

**Figure 6:**
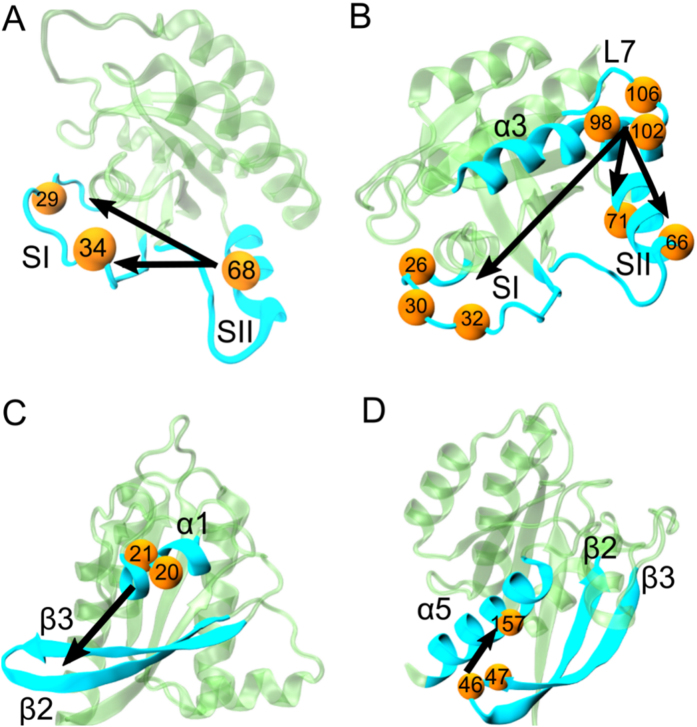
Causality relations in active K-Ras motions. Directionality in causal relationships is illustrated with arrows. Arrows start from driver residues and end at follower residues. Both residue types are represented with yellow spheres and marked with their residue numbers. The secondary structures they belong to are in turquoise. (**A**) R68 (SII) drives V29 and P34(SI). (**B**) E98 and R102 (α3) drive A66 (α2; SII). S106 (L7) drives Y71 (α2; SII). R102 (α3) drives N26 and Y32(SI). S106 (L7) drives D30. (**C**) ILE21-GLN22 (α1) drives β2-β3. (**D**) I46 and D47 (β2-β3) drive Y157 (α5).

Correlated motions of proteins often have a direction or causal relationship^30^. Correlations in the fluctuations of two residues indicate interaction, which is necessary for allosteric transitions. However, this is not sufficient for understanding the dynamic phenomenon completely since these symmetric correlations do not contain information on driver and follower relationships. To deduce causality, CTCs need to be analyzed. Our observation is supported by recent work^30^ that identified causality in correlated motions from MD simulations using an information theory measure of transfer entropy. This work, in turn, was built on a study by Schreiber, who introduced the *entropy transfer* concept for fluctuating environments^44^. We follow up on these ideas and introduce a new method to dissect dynamic correlations of all residue pairs of a protein to identify driver and follower residues. For this purpose, we evaluate strong time-delayed (**τ **= 5 ns) correlations between residue pairs. The strongest causal relations are as follows (Fig. 6).

#### SII motions drive SI in active K-Ras

**Figure 7:**
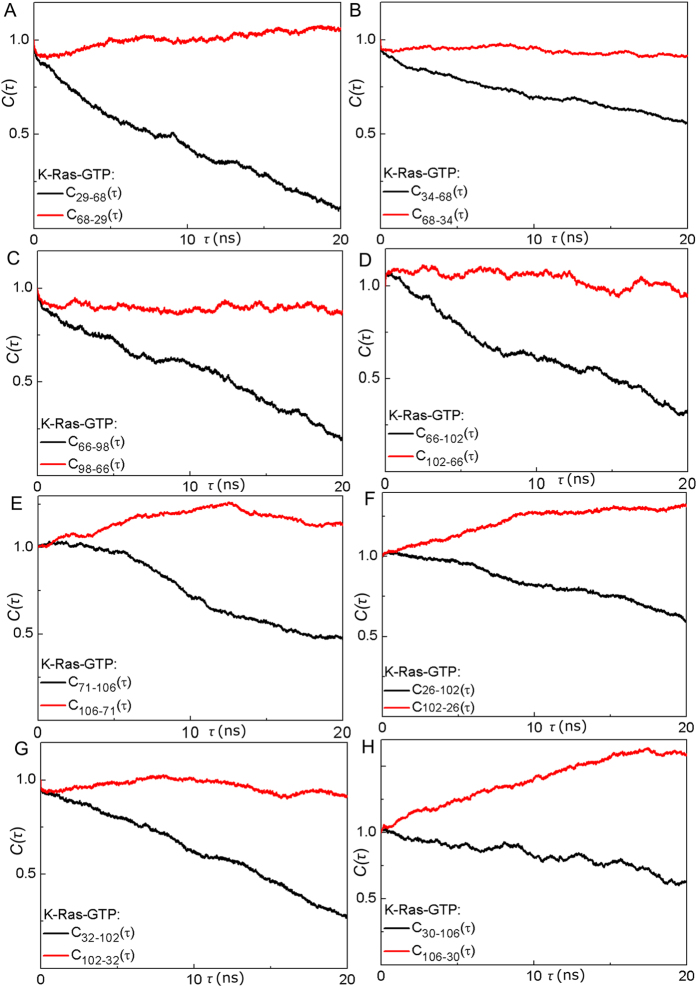
SII fluctuations drive SI fluctuations; α3-L7 motions drive switch region (SI & SII) motions in K-Ras-GTP. Red curves for Δ*R*_*i*_(*t*)Δ*R*_*j*_(*t* + *τ*) show that the fluctuations of residue *i* at time *t* affect the fluctuations of residue *j* at a later time *t* + *τ*. All correlations (*C(τ*)) are normalized with respect their value at zero (*C*(0)). (**A**) R68 (SII) drives V29(SI). (**B**) R68 drives P34(SI). (**C**) E98(α3) drives A66 (α2; SII). (**D**) R102 (α3) drives A66. (**E**) S106 (L7) drives Y71 (α2; SII). (**F**) R102 (α3) drives N26(SI). (**G**) R102 drives Y32(SI). (**H**) S106 (L7) drives D30(SI).

SI-SII relationship is better understood by examining residues that drive their motions throughout the trajectory. Our causality calculations show that SI is driven by SII (Figs 6A and 7). We present *CTC* plots of R68(SII) with V29(SI) (Fig. 7A) and with P34(SI) (Fig. 7B) for active K-Ras. Red curve shows that the fluctuations of R68 at time *t* affect the fluctuations of V29 at time *t* + *τ*. Fluctuation decay of K-Ras residues is in the order of 1 ns. The red curve persists for time periods that are an order of magnitude longer. The reverse does not show a significant correlation: V29 does not correlate with later fluctuations of R68. Previously, a study reported that SI loop at residues 29–34 swings into the water using V29 and P34 as hinges during Ras inactivation^37^. We improved on this information by calculating time-delayed correlations and identified that SII residues - especially R68 and D69- sustain active state conformation of SI by driving the motions of hinge residues V29 and P34. Another study also assessed the conformational transition of Ras from inactive to active state^45^, where displacement of SII triggers the active state transition and SI follows SII after a lag time of multiple nanoseconds. Dominance of SII region motions was also observed in several studies^46^,^47^. The nucleotide-bound form behavior is regulated by the relative arrangement of the two switches, rather than their individual conformations. We quantified this by verifying that SI fluctuations follow SII fluctuations in K-Ras-GTP. Since from an information theoretic point of view correlations are regarded as information sources, we conclude that information flows from SII to SI. The directionality originates from the differences in the characteristic decay times. The problem is one of dynamics within few nanosecond time periods. Disruption of this flow is expected to interfere with the switch mechanism function, which is the basis of K-Ras activity.

#### α3 and Loop 7 (L7) motions drive switch region (SI & SII) motions in active K-Ras

(Figure 6B) Fluctuations of the helical dimer interface residues of α3, E98 and R102^48^ drive fluctuations of A66 (α2; SII), as shown in Fig. 7C and D. Additionally, helical dimer interface residue S106 (L7) drives the motion of Y71 (α2; SII) (Fig. 7E). On the other hand, fluctuations of R102 (α3) and S106 (L7) drive SI residues N26, D30, Y32 (Fig. 7F to H).

Correlated motions of α2 and α3-L7 have been described in other studies, which also emphasized the necessity of understanding their effect on protein function^17^,^33^,^46^. We contribute to this knowledge by identifying their cause and effect relations. Furthermore, in previous studies, starting from the allosteric interaction between α2 and α3-L7, a novel ligand binding pocket, termed p3, which includes residues of L7 was defined and targeted for lead generation^17^,^49^,^50^. It was reported that ligand binding to p3 pocket weakens effector protein binding by allosterically stabilizing Ras effector binding site (SI). Another proposed allosteric mechanism is that ligand binding to p3 pocket changes the switch region conformation. Our results suggest that allosteric modulation of ligand binding may freeze the fluctuations of L7 and stabilize SI motions. This is based on our finding that motions of effector binding site (D30-Y32) are driven by S106 (L7).

#### β2 and β3 both drive and follow residue motions in active K-Ras

Causality calculations suggest the following information flow in fluctuations: ILE21-GLN22 (α1) drives β2-β3 (Figs 6 and S2); which drives Y157 (α5), Q61 (SII) and T74 (SII) (Figs 6 and S3, with details in Supplementary Table S4). Specifically, the differences in the characteristic decay times in Supplementary Fig. S3A,B demonstrate that information flows from β2-β3 to Y157 (α5). These findings improve on the previous observations of Abankwa *et al.* where they defined β2–β3 and α5 as a novel conformational switch^51^. Most importantly, we showed that Q61 (SII) motions follow E49 motions (β2-β3) (Supplementary Figure S3D). Abankwa *et al.* also observed that mutations in D47-E49 cause hyperactive Ras. Our findings support this too by showing that fluctuations of E49 of the wild type cause fluctuations of the catalytic residue Q61 within SII, whose proper positioning is essential for effective catalysis^52^. Based on these results, we suggest that mutations in D47-E49 region may alter E49 fluctuations that cause improper Q61 fluctuations. Therefore, GTP catalysis is disrupted which results in constituently active K-Ras.

## Conclusions

We present a novel approach that combines several distinct analysis methods to quantify in detail dynamics of GTP and GDP bound K-Ras, for which a significant amount of experimental and theoretical data already exists in the literature to test our predictions. Oncogenic K-Ras is a high priority drug target in cancer treatment. To develop new direct inhibitors that selectively bind to mutant K-Ras conformations while sparing those of WT K-Ras, it is necessary to first understand the dynamic activity of the WT protein in detail. To evaluate the nucleotide binding dependent changes in K-Ras stability, we used stiffness and RMSF calculations and proved that GTP binding rigidifies and hence stabilizes K-Ras motions. These results are in agreement with previous experimental and computational K-Ras studies^35^,^53^,^54^.

Our calculations that use stiffness, RMSF and correlation graphs (Figs 1 and 2, S1) confirm that GTP-binding increases K-Ras stiffness and thereby decreases fluctuation amplitudes, leading to distinct correlation patterns. These striking changes in GTP-bound-K-Ras dynamics enable its GTPase activity. Note that this nucleotide exchange is the first step in active to inactive transition^55^,^56^,^57^,^58^,^59^,^60^,^61^,^62^. Overall, our results support the well-established allosteric nature of K-Ras activation^59^, which has been suggested to play an important role in GTPase activity^63^. Although correlated fluctuations are necessary for allosteric information flow, their longer correlation decay times are also of crucial importance for complete allosteric transition. We calculated time-dependent autocorrelations of fluctuation vectors between residue pairs and discovered that correlations of K-Ras-GTP are stronger and persist for longer correlation times during simulations. Their persistency may allow complete allosteric information flow in K-Ras-GTP.

We broadened our analysis to quantify causality in allosteric regulation of K-Ras function. The most important results from our study are on causality. We applied a simple but powerful method that we defined as *CTC* into protein dynamics. To understand K-Ras dynamics, we investigated whether fluctuations of any residue caused fluctuations of another. Our results revealed the information flow in K-Ras switch mechanism and that SII fluctuations drive SI fluctuations. This prediction is an essential validation of our approach since the dominance of SII motions over SI motions was observed in previous experimental and computational studies^46^,^47^. Surprisingly, in addition to the canonical switch mechanism, our algorithm also revealed causality relations in the novel switch mechanism that includes β2–β3 and α5, where β2–β3 motions drive α5. Moreover, fluctuations of α3-L7 drive fluctuations of SI and SII. Interestingly, previous studies reported that Ras effector binding site (SI) is allosterically stabilized by ligand binding into a novel pocket that includes L7^17^,^49^,^50^. Our results explain the allosteric effect of ligand binding on SI motions by showing the information flow from L7 to SI.

Note that functionally, the identified driver and follower sites do not show an enrichment trend in oncogenic mutations observed in human cancers (from 2266 missense K-Ras mutations observed in all cancers within cBioPortal www.cbioportal.org on July 28, 2016). However, the motions of the second most frequently mutated residue in cancer patients (113/2266 missense mutations), Q61 (SII region), are driven by those of E49 (β3). While there are no known oncogenic mutations of residue E49, experiments have shown that mutating E49 leads to hyperactive Ras^51^, similar to the effects of Q61 mutations.

Our long-term goal is to discover small molecules that can eliminate unfavorable deviations from intrinsic K-Ras dynamics due to oncogenic mutations. While our method is directly applicable to study the effects of oncogenic mutations or effector protein binding on K-Ras dynamics, each one of these investigations first requires a deep understanding of the causal relationships in intrinsic K-Ras motions. The detailed results we present here will serve as a reference point for such studies. The computational tools we introduce in the present work are also easily applicable to the analysis of simulation data from different proteins to understand causality in their allosteric regulations, which can then similarly be utilized in drug discovery. From this perspective, our approach has the potential to set a novel paradigm for drug design by directing attention to changes in protein dynamics. The latter is in close relation to changes in protein function whose restoration to normal is the target of all drug design activities.

## Methods

### MD Simulations

We performed all-atom MD simulations for both Mg^+2^GDP- and Mg^+2^GTP-bound K-Ras. Crystal structure of K-Ras-GDP was retrieved from Protein Data Bank (PDB ID: 4OBE) and K-Ras-GTP structure was obtained by changing GDP to GTP by adding a phosphate group to GDP using Discovery Studio 4.5 software, (DS)^64^. Then, the complex was optimized with Clean Geometry tool of DS. This geometry optimization tool uses a fast, Dreiding-like forcefield to improve the geometry of the selected atoms and results in an approximate 3D structure. We solvated each protein in a TIP3P water box with 12 Å buffering distance. We applied periodic boundary conditions and added ions to neutralize the system. We used a 2 fs time-step with a 12 Å cutoff for Van der Waals interactions and full particle-mesh Ewald electrostatics with 1 Å grid spacing and direct space tolerance of 10^−6^. We carried out all computations in N, P, T dynamics procedure. System temperature was kept constant at the physiological value of 310 K using Langevin dynamics with a damping coefficient of 2 ps^−1^. Constant pressure of 1 atm was maintained by The Nose-Hoover Langevin piston method with a 200 fs piston period and 100 fs decay time. We used NAMD 2.10^65^ with AMBER ff99SB^66^ and general amber force fields (GAFF)^67^. We obtained parameters of GTP and GDP (see Supplementary Methods). The initial system energy was first minimized for 10,000 steps, followed by 10,000 steps for equilibration. After equilibration, we performed 300 ns simulations. Atomic coordinates 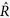 of all atoms were saved every 1 ps. To eliminate all rotational and translational motions, we aligned the trajectories to the initial structure by using VMD software 1.9.2^68^. We visualized trajectories using VMD. Additionally, to test whether WT K-Ras-GTP complex approached the active (close) state, we monitored the first 100 ns-trajectories of both GTP- and GDP-bound complexes with VMD. We observed that the active site residues obtain relatively close conformations in WT K-Ras-GTP. We calculated distance distributions of several residue pairs that flank GTP and observed that they were in support of the relatively close active site conformation.

### Stiffness

We quantified nucleotide-bound K-Ras stiffness using a statistical thermodynamics interpretation of fluctuation correlations^69^. We assumed that the interaction between two fluctuating residues *i* and *j* can be represented by a spring, where the spring constant follows from the Gaussian Network Model (GNM)^36^: *k*_*ij*_ = (*k*_*β*_*T*)/(〈(Δ*R*_*i*_)^2^〉 − 2〈(Δ*R*_*i*_Δ*R*_*j*_)〉 + 〈(Δ*R*_*j*_)^2^〉) where Δ*R*_*i*_ is the instantaneous fluctuation of one end of the rod, Δ*R*_*j*_ is the fluctuation of the other end (Details in Supplementary Methods), *k*_*B*_is the Boltzmann constant and *T* is the absolute temperature. The spring constant has dimensions of force/length. In GNM spring definition, each residue *i* is attached to *N* − *1* other residues via *N* − *1* springs^36^. Thus, how stiffly a residue *i* is attached to a protein can be quantified by 
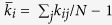
 where 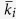 is the mean spring constant for a residue *i*. For stiffness estimates of the complete complexes, we define an overall stiffness parameter *k*_*overall*_ by the expression 
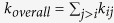
. To estimate the stiffness differences in active versus inactive K-Ras, we calculated 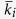 for each residue and *k*_*overall*_ for the protein for both states.

### Stability

We defined the stability of an interacting system of residues as the joint state of reduced RMSF and increased interaction stiffness. RMSF relates to the magnitude of fluctuations of individual residues and stiffness relates to the distance between two residues and therefore they are two independent quantities. (For details see Supplementary Methods). A small RMSF and a high stiffness denote increased stability.

### Distance distributions between residue pairs

We calculated the distance between two residues (*i*, *j*) as 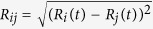. Residue pair distance distributions *W(R*_*ij*_) were calculated by dividing the maximum distance between the pair into small bins and counting the number of observed distances in each bin. All distributions were normalized.

### Time independent correlations (cross-correlation coefficient map)

Correlations intrinsic to K-Ras structure are defined by the cross-correlation coefficient map, *C(ΔR*_*i*_*, ΔR*_*j*_):


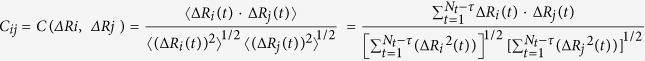


where ∙ denotes the dot product. Correlation varies between −1 and 1. If motions of two atoms are independent, then 〈Δ*R*_*i*_(*t*) ⋅ Δ*R*_*j*_(*t*)〉 = 0 and *C*_*ij*_ = 0. If the atoms always move in parallel in the same direction, then they are perfectly positively correlated, and *C*_*ij*_ = 1. If they always move in parallel in opposite directions, they are perfectly negatively correlated, and *C*_*ij*_ = −1. Cross-correlation coefficients lie in the range of −1 ≤ *C*_*ij*_ ≤ 1.

### Time delayed correlations, mobility and causality

Time-delayed correlation of two fluctuations is defined by:


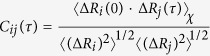


where χ denotes that the average is a conditional average calculated according to


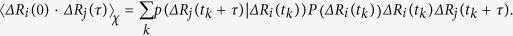


Here, *p*(Δ*R*_*j*_(*t*_*k*_ + *τ*)|Δ*R*_*i*_(*t*_*k*_))denotes the conditional probability of observing Δ*R*_*j*_(*t*_*k*_ + *τ*) at time *t*_*k*_ + *τ*, given Δ*R*_*i*_(*t*_*k*_) at time *t*_*k*_.

Similarly, if indices are exchanged, then *C*_*ji*_(*τ*) represents the correlations of *ΔR*_*i*_at time *t* + *τ* with earlier *ΔR*_*j*_ values at time *t*. If the fluctuations of residue *i* drive the fluctuations of residue *j*, then *C*_*ij*_(*τ*) > *C*_*ji*_(*τ*). If *C*_*ji*_(*τ*) > *C*_*ij*_(*τ*), residue *j* drives residue *i* because the fluctuation *ΔR*_*j*_ at time *t* is correlated with future fluctuations of *ΔR*_*i*_. However, at *τ* = 0, the equality *C*_*ij*_(0) = *C*_*ji*_(0) holds.

Note that time-delayed autocorrelation *C*_*ii*_(*τ*) is the correlation of the trajectory with its own past and future coordinates. If autocorrelation is large, it can correspond to a specific form of “persistence”, a tendency for a system to remain in the same state from one observation to the next.

## Additional Information

**How to cite this article**: Vatansever, S. *et al.* Intrinsic K-Ras dynamics: A novel molecular dynamics data analysis method shows causality between residue pair motions. *Sci. Rep.* **6**, 37012; doi: 10.1038/srep37012 (2016).

**Publisher’s note**: Springer Nature remains neutral with regard to jurisdictional claims in published maps and institutional affiliations.

## Supplementary Material

Supplementary Information
