# Supplementary material for: Intrinsic K-Ras dynamics: A novel molecular dynamics data analysis method shows causality between residue pair motions
Source: Sci Rep. 2016 Nov 15;6:37012. doi: 10.1038/srep37012 (PMC5109477; doi:10.1038/srep37012)
Supplement: Supplementary Information [file srep37012-s1.pdf]

SUPPLEMENTARY INFORMATION

Intrinsic K-Ras dynamics: A novel molecular dynamics data analysis method shows causality between residue pair motions

Sezen Vatansever[1,2,3], Zeynep H. Gümüş[2, 3*], Burak Erman[1*]

[1]Department of Chemical and Biological Engineering, College of Engineering, Koç University, Rumelifeneri Yolu, 34450, Sarıyer, Istanbul, Turkey

[2]Department of Genetics and Genomics, [3]Icahn Institute for Genomics and Multiscale Biology, Icahn School of Medicine at Mount Sinai, New York, NY 10029

*Co-correspondents: berman@ku.edu.tr (B.E.) and zeynep.gumus@mssm.edu (Z.H.G.)

Supplementary Figures

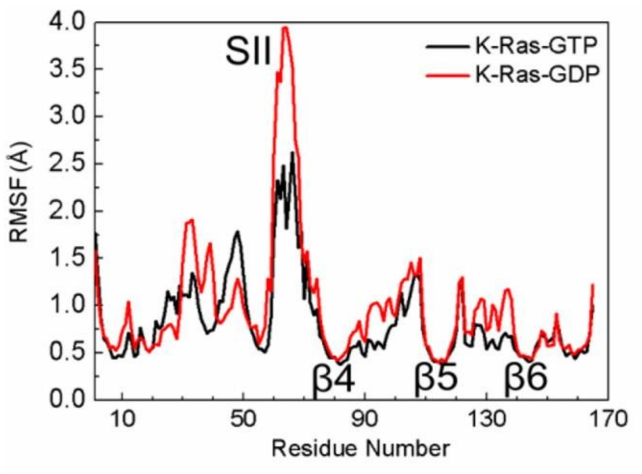

Figure S1. Comparison of RMSF values of K-Ras motion in active form with inactive form. RMSF values of active form (black) and inactive form (red).

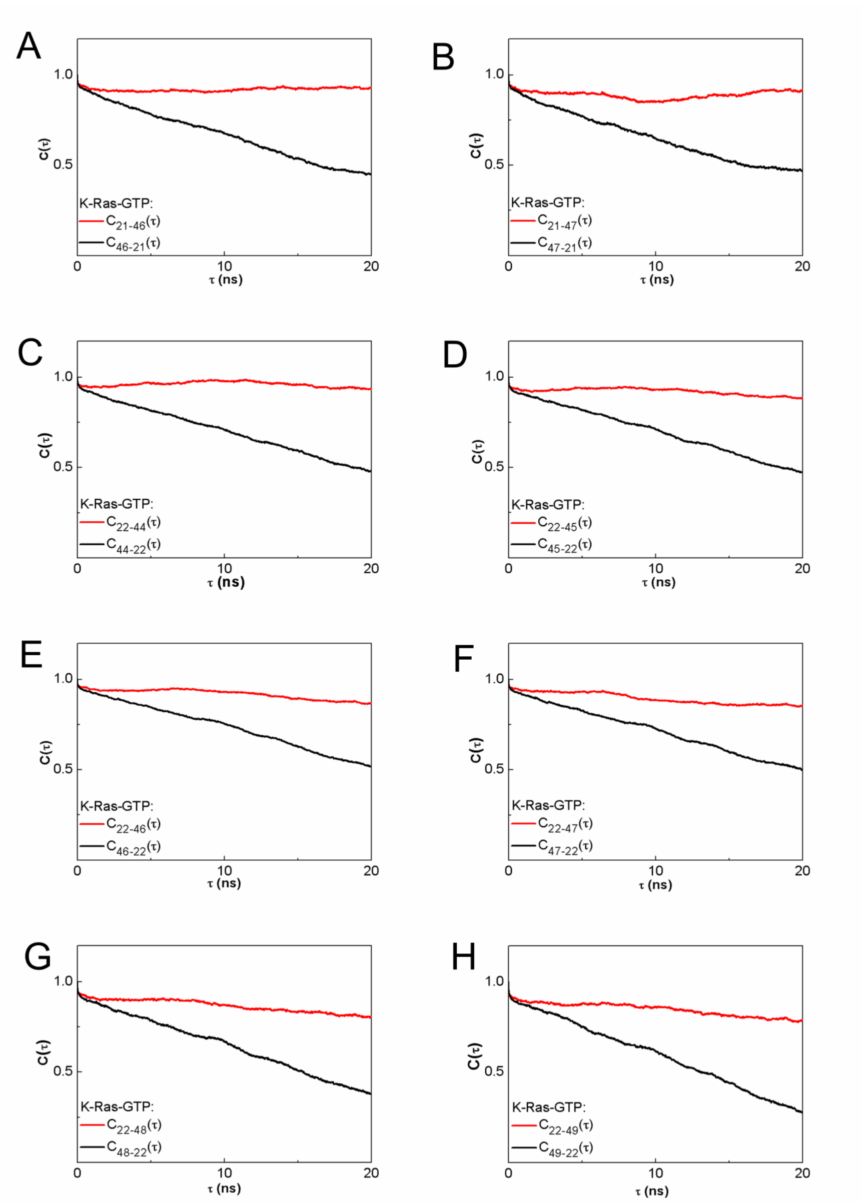

Figure S2. Fluctuations of ILE21-GLN22 (α1) drive the fluctuations of β2-β3. The red curves are for $\langle \Delta Ri(t) \Delta Rj(t+\tau) \rangle$ and show that the fluctuations of residue *i* at time *t* affect the fluctuations of residue *j* at a later time $t+\tau$. (A), (B) I21 drives I46 and D47, respectively. (C)-(H) Q22 drives V44, V45, I46, D47, G48 and E49, respectively.

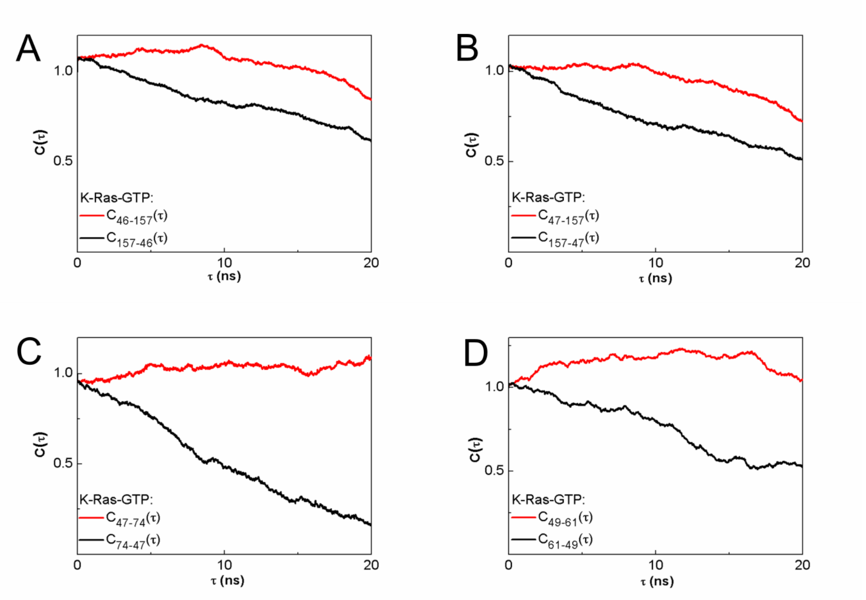

Figure S3. Fluctuations of β2-β3 region drive the fluctuations of Y157 (α5), Q61 (SII) and T74 (SII). The red curves are for $\langle \Delta Ri(t)\Delta Rj(t+\tau)\rangle$ and show that the fluctuations of residue *i* at time *t* affect the fluctuations of residue *j* at a later time $t+\tau$. (A), (B) Y157 follows I46 and D47, respectively. (C) D47 drives T74. (D) E49 drives Q61.

Supplementary Tables

Table S1. Categorization of $\bar{k}_1$ values

$\bar{k}_1$ RANGE	$\bar{k}_1$ VALUES (kcal/mol·A^2)		CATEGORY
	K-Ras-GTP	K-Ras-GDP	
%100	1.28 (Max)	1.08 (Max)	The Highest
%90	≥ 1.16	≥ 0.97	
%80	≥1.04	≥ 0.86	High
%10<br>%100	≤ 0.19<br>Min 0.08	≤ 0.14<br>Min 0.03	The Smallest

Table S2. Categorization of residues according to their mean spring constant $\bar{k}_1$ values for K-Ras-GTP (left) and K-Ras-GDP (right).

	K-Ras-GTP		K-Ras-GDP	
	Region	Residues	Region	Residues
The Most Rigidly Attached	β4	G77-I84	β4	G77-I84
	β5	P110-N116	β5	P110-N116
	β6	P140-T144	β6	P140-T144
Rigidly Attached	β1	L6-G10	β1	L6-G10
	P-loop	V14-G15	P-loop	V14-G15
	α5	F156-V160	α5	F156-V160
The Most Flexibly Attached	SII	G60-M67	SI	D30-P34
			SII	A59-R68

Table S3. Comparison of the average number of hydrogen bonds formed throughout the simulation between the nucleotide and K-Ras.

	Average number of H bonds throughout the simulations	
Residue of K-Ras	GTP	GDP
SER17	0.45	0.20
ASP30	1.00	0.61

Table S4. The β2-β3 region acts as both the driver and the follower in active K-Ras.

DRIVER RESIDUE(S)		FOLLOWER RESIDUE(S)	
RESIDUE(S) NAME	REGION	RESIDUE(S) NAME	REGION
I21-GLN22	α1	Q43-L53	β2- β3
I46-CYS51	β2- β3	Y157	α5
I46-GLY48	β2- L3	T74	α2; SII
E49	β3	Q61	SII

Supplementary Methods

Parametrization of GTP and GDP

The procedure consisted of two parts: (i) charge derivation; and (ii) preparation of the coordination and parameter files for the complexes. Charge derivation was divided into three sequential steps. First, we optimized the initial GTP and GDP structures by using QM program Gaussian 03 with base 6-31G* and level HF. Second, we performed Molecular Electrostatic Potential (MEP) calculations using Gaussian 03 with base cc-pVTZ and the Density Functional Theory (DFT) method B3LYP in continuum solvent. The third step was Restrained ElectroStatic Potential (RESP) charge derivation[1]. We used RESP program available under AMBER[2]. We automated the calculations using the programs RED-vIII, Ante_RED and Antechamber[2-4]. The RESP ESP charge Derive program (R.E.D.) sequentially executed these three steps by interfacing Gaussian and RESP programs, and allowed the automatic derivation of RESP and ESP charges for GTP and GDP. Ante_RED was useful to prepare input files for Gaussian. We used RED-vIII to perform MEP calculations and RESP charge derivations. Antechamber wrote out additional force field files (frcmod file) of molecules with missing parameters. For basic model building and Amber coordinate and parameter/topology file creation we used the LEaP module of AMBER 14 package[5].

Calculation of fluctuations

MD trajectories describe the time evolution of a system and are defined by the position vector $R_i(t)$ *of* every atom *i* at every time point *t* where $1 \leq t \leq N_T$ and N_T is the total number of *t* time points of the trajectory. The time average $\bar{R_i}$ position of each atom *i* is then defined as $\bar{R}_i = \sum_t^{N_T} R_i\,(t)/N_T$. The instantaneous fluctuation $\Delta R_i(t)$ of the position vector of the *i*th atom is defined as $\Delta R_i(t) = \Delta R_i(t) - \bar{R}_i$. The root mean squared fluctuation (RMSF) of a residue is

$\left\langle \left(\Delta R_i \right)^2 \right\rangle^{0.5}$, where the angular brackets show the time average of the quantity enclosed. The RMSF of each atom is proportional to its Debye-Waller (B-factor) measured experimentally.

Supplementary References

1 Bayly, C. I., Cieplak, P., Cornell, W. D. & Kollman, P. A. A Well-Behaved Electrostatic Potential Based Method Using Charge Restraints for Deriving Atomic Charges - the Resp Model. *J Phys Chem-Us* **97**, 10269-10280, doi:DOI 10.1021/j100142a004 (1993).

2 Dupradeau, F. Y. *et al.* The R.ED. tools: advances in RESP and ESP charge derivation and force field library building. *Phys Chem Chem Phys* **12**, 7821-7839, doi:10.1039/c0cp00111b (2010).

3 Wang, J. M., Wang, W. & Kollman, P. A. Antechamber: An accessory software package for molecular mechanical calculations. *Abstr Pap Am Chem S* **222**, U403-U403 (2001).

4 Vanquelef, E. *et al.* R.E.D. Server: a web service for deriving RESP and ESP charges and building force field libraries for new molecules and molecular fragments. *Nucleic Acids Res* **39**, W511-517, doi:10.1093/nar/gkr288 (2011).

5 D.A. Case, V. B., J.T. Berryman, R.M. Betz, Q. Cai, D.S. Cerutti, T.E. Cheatham, III, T.A. Darden, R.E. Duke, H. Gohlke, A.W. Goetz, S. Gusarov, N. Homeyer, P. Janowski, J. Kaus, I. Kolossváry, A. Kovalenko, T.S. Lee, S. LeGrand, T. Luchko, R. Luo, B. Madej, K.M. Merz, F. Paesani, D.R. Roe, A. Roitberg, C. Sagui, R. Salomon-Ferrer, G. Seabra, C.L. Simmerling, W. Smith, J. Swails, R.C. Walker, J. Wang, R.M. Wolf, X. Wu and P.A. Kollman. *AMBER 14*, 2014).
